# Recyclable, Antibacterial, Isoporous Through-Hole Membrane Air Filters with Hydrothermally Grown ZnO Nanorods

**DOI:** 10.3390/nano11123381

**Published:** 2021-12-13

**Authors:** Yong Ho Choi, Moon-Ju Kim, Jia Lee, Jae-Chul Pyun, Dahl-Young Khang

**Affiliations:** Department of Materials Science and Engineering, Yonsei University, Seoul 03722, Korea; yhyhyh825@naver.com (Y.H.C.); moonjukim@yonsei.ac.kr (M.-J.K.); jia501@naver.com (J.L.); jcpyun@yonsei.ac.kr (J.-C.P.)

**Keywords:** ZnO nanorods, antibacterial, isoporous membrane, air filter, reusability

## Abstract

Reusable, antibacterial, and photocatalytic isoporous through-hole air filtration membranes have been demonstrated based on hydrothermally grown ZnO nanorods (NRs). High-temperature (300~375 °C) stability of thermoset-based isoporous through-hole membranes has enabled concurrent control of porosity and seed formation via high-temperature annealing of the membranes. The following hydrothermal growth has led to densely populated ZnO NRs on both the membrane surface and pore sidewall. Thanks to the nanofibrous shape of the grown ZnO NRs on the pore sidewall, the membrane filters have shown a high (>97%) filtration efficiency for PM_2.5_ with a rather low-pressure (~80 Pa) drop. The membrane filters could easily be cleaned and reused many times by simple spray cleaning with a water/ethanol mixture solution. Further, the grown ZnO NRs have also endowed excellent bactericidal performance for both Gram-positive *S. aureus* and Gram-negative *S. enteritidis* bacteria. Owing to the wide bandgap semiconductor nature of ZnO NRs, organic decomposition by photocatalytic activity under UV illumination has been successfully demonstrated. The reusable, multifunctional membrane filters can find wide applications in air filtration and purification.

## 1. Introduction

Threats to human health caused by microorganisms in the environment are emerging as a major problem worldwide. In particular, airborne particulate matter (PM) contains not only inorganic particles and liquid droplets, but also bioorganic particles such as bacteria, fungi, and viruses, and it is known that significant parts of inhalable microorganisms can cause infectious diseases, respiratory diseases, and allergies [1,2]. Bacteria captured directly in the filter, or pathogenic microorganisms that have grown on the surface of the filter under humid environments due to breathing and saliva, can cause secondary air pollution [3,4,5]. Moreover, microorganisms accumulated in the filter can cause a decrease in the performance and life of the filter. Meanwhile, since the SARS-CoV-2 virus, which has emerged as a global issue now, is capable of human-to-human transmission, the importance of facial mask filters to block the transmission is emerging [6,7,8]. Accordingly, in addition to the high PM removal efficiency and low-pressure drop required for conventional air filters, the need for multifunctional air filters with excellent antibacterial performance is demanding [9].

To endow bactericidal properties to filtration membranes, special antibacterial agents are usually added or grafted into membranes. Well-known antibacterial agents include metal nanoparticles, organics, and wide bandgap metal-oxide semiconductors. Metal nanoparticles such as Ag [10,11,12] and Cu [13,14] have been successfully applied as antibacterial additives, either in direct composite with filtration polymeric materials or as surface coatings. However, nanotoxicity of metal nanoparticles and their leaching behavior evoke concerns in certain applications [15,16]. Organic antimicrobial agents, such as quaternary ammonium salts [17,18,19], chitosan [20,21], capsaicin [22], and N-halamine (nitrogen atom with direct linkage to halogens) [18,21], suffer from inefficient performance, biotoxicity, or are irritant to human skin [18]. Among metal-oxide-based antibacterial agents such as TiO_2_ [23] and WO_3_ [24], ZnO [25] has been known as antibacterial and non-toxic (or, biocompatible) to humans. In addition, various forms of ZnO nanostructures can easily be synthesized by the hydrothermal approach [26,27], which is a scalable and low-temperature solution process. The bactericidal mechanisms of ZnO are not fully understood yet, but suggested mechanisms include (1) the generation of reactive oxygen species (ROS), (2) the release of Zn^2+^ ions, and (3) mechanical damage of the cell wall.

Among various types of air filter, fibrous filters [28,29,30,31,32,33,34] are the most popular, usually formed by the random stacking of polymeric micro/nanofibers produced by melt-blowing or electrospinning. The large surface to volume ratio of the fibrous filters leads to high filtration efficiency with low-pressure drop. For antibacterial fibrous filters, bactericidal agents discussed above are incorporated or grafted into/onto polymeric matrices. However, the mechanical strength of fibrous filters is usually insufficient, and thus there exists a trade-off between efficiency and pressure drop: thicker filters ensure high efficiency and mechanical stability, but with a high-pressure drop. Further, the electrospinning process needs to apply very high (few tens of kV) voltage and employs volatile organic solvents, which are potential hazards to operators and the environment. The process is serial in nature, which takes a long time to cover a large area. More importantly, it is not easy to clean the PMs trapped deep inside the nanofiber network, disabling reusability.

In this work, we have demonstrated recyclable and antibacterial isoporous through-hole membranes as high-performance air filters. The isoporous through-hole membranes were prepared by a simple, parallel, and cost-effective soft lithographic method. Thanks to the high thermal stability of the membranes based on thermoset polyurethane acrylate (PUA) resin [35], the seed for hydrothermal ZnO nanorods’ (NRs) growth could be directly formed on the membrane surface by high-temperature annealing. After the growth, ZnO NRs have been formed on the sidewall of pores, as well as the membrane surface. The grown ZnO NRs greatly enhanced the filtration efficiency (>97%) while minimally increasing the pressure drop (~ 80 Pa), due to the fibrous shape of the NRs grown on the sidewall of pores. Additionally, the PM-contaminated ZnO NR-grown membrane filters can be reused multiple times after simple spray cleaning with a water/ethanol mixture. In addition, the grown ZnO NRs endow the membrane filters with bactericidal activity for both Gram-positive *Staphylococcus aureus* (*S. aureus*) and Gram-negative *Salmonella enteritidis* (*S. enteritidis*). Further, organic decomposition by photocatalytic activity under UV illumination has been verified with the ZnO NR-grown isoporous through-hole membrane filters, thanks to the semiconductor nature of the grown ZnO NRs. The proposed membrane filters with multi-functionality may find broad applications in air filtration and purification.

## 2. Materials and Methods

### 2.1. Materials

SU-8 (MicroChem) was used as a negative photoresist. Polydimethylsiloxane (PDMS) base resin and curing agent (Sylgard 184) were purchased from Dow Corning Company. The UV-curable polyurethane acrylate resin (MINS-311RM) was purchased from MCNET Co. Ltd., Korea. Zinc acetate dihydrate (Zn(CH_3_COO)_2_·2H_2_O), ethanolamine (NH_2_CH_2_CH_2_OH), zinc nitrate hexahydrate (Zn(NO_3_)_2_·6H_2_O), and hexamethylenetetramine (HMTA, C_6_H_12_N_4_) were purchased from Sigma-Aldrich and used as-received. Luria-Bertani (LB) broth was purchased from Duchefa, The Netherlands. Phosphate-buffered saline (PBS) was purchased from Curebio, Korea.

### 2.2. Isoporous Through-Hole Membrane Preparation

Master molds were prepared by photolithography (MJB6, SUSS MicroTec) using SU-8 as a negative photoresist. The SU-8 was spun on cleaned Si as a substrate. The photomask has a dots array, having diameter/space of 7 and 3 μm, respectively, which has led to a holes array on SU-8 resist upon exposure and development. The elastomeric PDMS stamp was replicated from the holes array-patterned SU-8 master. A mixture of base resin and curing agent (10:1 *w/w*) of PDMS was placed in a vacuum desiccator for 1 h to remove trapped air bubbles. Then, the degassed pre-polymer mixture was poured onto the SU-8 master and cured in an oven at 80 °C for 3 h. After the cured PDMS stamp was peeled off the master, it was cut into a 1.9 × 1.9 cm^2^ size. Then, the PDMS stamp had a cylindrical pillars array protruding from its surface. For the preparation of isoporous through-hole membranes, UV-curable resin (MINS-311RM) was dropped onto the pillar-patterned PDMS stamp, and covered with a thin wrap film and scraped with a solid edge to remove excessive resin. After UV curing for 1 h, a free-standing isoporous through-hole membrane can be peeled-off the PDMS stamp after removing the wrap film. The thickness of the membrane is largely determined by the thickness of the SU-8 photoresist, which was ~3 μm in this work. More details on the membrane fabrication can be found elsewhere [36].

### 2.3. ZnO Seed Layer

The seed layer solution was prepared by dissolving zinc acetate dihydrate (0.1 M) and ethanolamine (0.1 M) in ethanol and stirring it for 1 h. For the spin coating, the free-standing isoporous through-hole membrane was laminated onto a flat cured slab of PDMS as a carrier substrate, and the exposed surface of the membrane was treated with a brief O_2_ plasma (30 W/3 min) for uniform coating. The seed layer solution was spun onto the membrane at 1000 rpm for 30 s, and then the sample was dried on a hotplate at 70 °C for 8 min. To achieve a good coverage of the ZnO seed layer on the membrane surface/pore sidewall, the seed-coating process was repeated three times, with drying on a hotplate after each spin-coating. Finally, the membrane was separated from the carrier substrate and annealed in a furnace to form a ZnO seed layer at high temperature, in the range of 300~375 °C. Note here that the pore size and spacing, i.e., porosity, of isoporous through-hole membranes was changed after the annealing due to thermal shrinkage.

### 2.4. Hydrothermal Growth of ZnO Nanorods

ZnO seed-coated membranes were immersed in a 70 mL aqueous solution of equimolar (20 mM) zinc nitrate hexahydrate and HMTA in a heating mantle. The hydrothermal growth was carried out at 90 °C for 5 h. Subsequently, the membranes that grew ZnO NRs were washed with a copious volume of de-ionized (DI) water before drying on a hotplate at 100 °C for 10 min.

### 2.5. Characterizations

A scanning electron microscope (SEM; S-5000, Hitachi, Chiyoda City, Tokyo, Japan) was used for the imaging of the membrane surface. 

### 2.6. PM Filtration Test

PM particles were generated by burning incense. The smoke PM particles have a size distribution in the range from <300 nm to >10 µm, and most particles have a diameter of <1 µm [32,37]. The generated PM was then diluted with fresh air to control the hazardous pollution level of PM index > 300. The light scattering-based fine dust meters (JSMY-2000, 3M, Changseong SCO, Gyeonggi-do, Korea) were used to detect PM concentrations in the inlet and outlet chambers, respectively, and the filtration efficiency was calculated based on both readings. The pressure drop and flow velocity were measured using a differential pressure gauge (testo 510, Testo) and anemometer (testo 405-V1, Testo), respectively. Supplementary Figure S1 shows the schematic drawing of our experimental set-up.

### 2.7. Antibacterial and Photocatalytic Tests

Gram-negative *S. enteritidis* and Gram-positive *S. aureus* bacteria were used for the antibacterial tests. Cultured bacterial cells were centrifuged at 8000× *g* for 3 min, 3 times, and re-suspended in phosphate-buffered saline (PBS). The optical densities (ODs) of the two bacterial cultures at 600 nm (OD_600_) were determined and adjusted to 0.5 using normal saline. After both pristine and ZnO NR-grown membranes were cut into 0.5 × 0.5 cm^2^ and autoclaved for sterilization, the membrane samples were placed at the bottom of 96-well plates. Each 96-well plate was filled with bacterial culture of 350 μL (OD_600_ = 0.5). The absorbance of the cells from each well was measured at 600 nm every 3 h, up to 6 h, using a microplate reader (Versamax, Molecular Devices). For fluorescence microscopy (BX51, Olympus) imaging, the cells were stained with acridine orange, which is nucleic acid-selective fluorescent dye with cationic properties. The dye can permeate into the cell membrane, leading to an interaction with dsDNA and RNA or ssDNA via intercalation and electrostatic interaction, respectively. The dyes emit green fluorescence (526 nm) for live cells and red fluorescence (650 nm) for dead ones.

Under UV-excitation, ZnO NRs form oxidative *h*^+^ at the valence band and reductive *e*^-^ at the conduction band. The formed carriers can oxidize water by *h*^+^ and reduce water or oxygen by *e^−^*, leading to the formation of ROS, such as ·O_2_^−^, ·OH, and H_2_O_2_. For photocatalytic activity of ZnO NR-grown membranes, the optical density (at 650 nm) of methylene blue (MB) solution was monitored. As a control, the pristine membrane was also dipped in MB solution and the OD_650_ of the solution was traced as a function of time.

## 3. Results and Discussion

### 3.1. Morphology of ZnO NR-Grown Membranes

**Figure 1:**
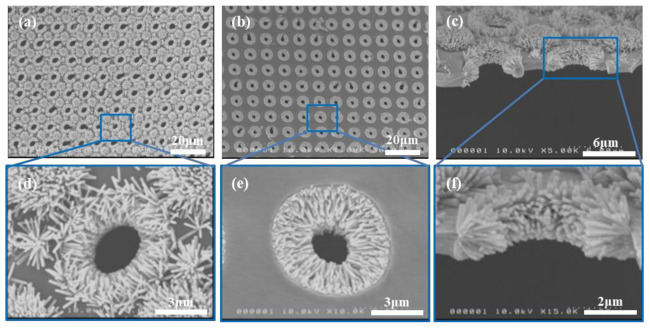
SEM images of isoporous through-hole membranes with hydrothermally grown ZnO NRs. The seed was formed by annealing at 325 °C. (**a**–**c**) Top, bottom, and cross-sectional view SEM images of the membranes, respectively, and their respective magnified images (**d–f**).

The thermal stability of our isoporous through-hole membranes enabled us to prepare the seed layer directly on the membrane surface for the following hydrothermal growth of ZnO NRs. Otherwise, seeds for the growth should be prepared by expensive vacuum-based processes, such as ALD or sputtering [38,39,40]. Figure 1 presents the SEM images of the membrane surface after the hydrothermal growth of ZnO NRs. After the growth, as shown in Figure 1a, the membrane surface is covered with densely populated ZnO NRs grown largely in the vertical direction. Upon magnification (Figure 1d), however, some regions of the membrane surface have been found to have no ZnO NRs, except the diagonal centers and rim/sidewall of pores. The observed non-uniform growth of ZnO NRs on the top surface of the membrane was due to the non-uniform seed layer distribution on it: diagonal centers between four neighboring pores have been found to have a thick seed layer, due to the non-flat bottom surface of the PDMS stamp used (Supplementary Figure S2). Areas that have a very thin or no seed layer seem to not have enough ZnO nuclei to induce the growth of ZnO NRs.

### 3.2. Apparent Porosity and Filtration Performance of ZnO NR-Grown Membranes

It should be noted that the membrane was laminated onto a carrier PDMS substrate for the spin-coating of the seed solution, as mentioned above. In other words, the bottom surface of the membrane that is in intimate contact with the PDMS carrier substrate does not have any seed at all. Figure 1b shows the SEM image of the bottom surface of the membrane, which shows that the bottom surface is free of ZnO NRs. Upon magnification (Figure 1e), arrays of ZnO NRs were densely grown on the sidewall of pores in the perpendicular direction to the pore sidewall. This pore growth has significant implications on the filtration performance and will be discussed later. Figure 1c shows the cross-sectional SEM image of the membrane with grown ZnO NRs. The exploded view of the cross-section, as shown in Figure 1f, clearly shows the densely populated ZnO NRs array on the pore sidewalls. Note that the NRs grown on pore sidewalls are discrete entities, that is, they do not completely block the passage of PM-laden air flow. Rather, the ZnO NRs array plays quite a similar role to nanofibers in fibrous air filters. Therefore, the grown NRs array can greatly contribute to the enhanced filtration efficiencies, while its contribution to the increase of the pressure drop was marginal.

**Figure 2:**
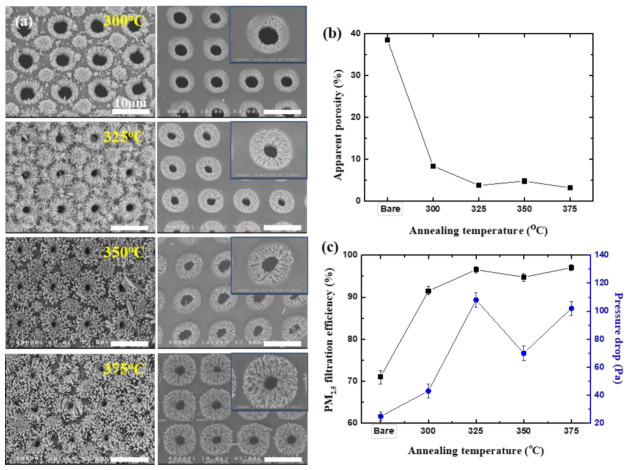
ZnO NR-grown isoporous through-hole membranes and their air filtration performances. (**a**) SEM images of membrane surfaces, top (**left** column) and bottom (**right** column) surfaces, respectively, prepared at different annealing temperatures after the spin-coating of the seed layer. (**b**) Apparent porosity and (**c**) filtration efficiency and pressure drop, respectively, for ZnO NR-grown membrane filters shown in (**a**).

**Figure 3:**
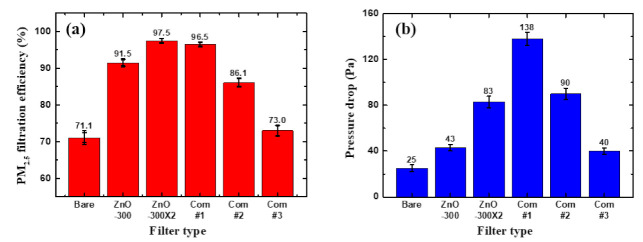
Comparison of filtration performances between different membrane filters, including some commercial ones. (**a**) Filtration efficiencies and (**b**) pressure drops, respectively.

Figure 2a shows the SEM images, both top (left column of Figure 3a) and bottom (right column) surfaces, of isoporous through-hole membranes with grown ZnO NRs. The annealing of seed solution was carried out at different temperatures (300~375 °C). As mentioned above, the high-temperature annealing for seed formation also led to the change in pore size and spacing due to thermal shrinkage of the PUA membrane (Supplementary Figures S3 and S4 for the porosity change of membranes with annealing temperature under different sample conditions, together with filtration performances of those membranes without ZnO NRs, such as efficiency and pressure drop). First of all, ZnO NRs’ growth on pore sidewalls has occurred at all annealing temperatures investigated in this work, from 300 up to 375 °C, and the NRs’ growth took place only on diagonal centers that have a thicker seed layer on the top surface of the membrane, as shown in Figure 1. Further, the ZnO NRs’ growth on pore sidewalls, which is critically important for filtration performance of the membranes, has been observed for all annealing temperatures. Although we did not precisely measure the porosities of ZnO NR-grown isoporous membranes, the apparent porosities (areal fraction of void) of the membranes shown in Figure 2a were estimated based on SEM images and plotted in Figure 2b. Compared to the porosity of the bare membrane without ZnO NRs (~40%), the apparent porosities of ZnO NR-grown membranes decreased a lot, down to 5~10%. The decrease in porosity will definitely lead to the enhanced filtration efficiency. Indeed, all those membranes having ZnO NRs on the sidewall of pores showed enhanced filtration efficiencies, 91~97%, as shown in Figure 2c, compared to a filtration efficiency of 60~70% for bare membranes without ZnO NRs (labeled as ‘Bare’ in Figure 2). At the same time, the increase in pressure drop for those membranes with ZnO NRs was marginal, 40~110 Pa, as shown in Figure 2c, as expected. Thanks to the fibrous geometry of the ZnO NRs, they do not severely block the air flow, which results in the marginal increase in the pressure drop. It should be noted that the filtration performances of as-prepared isoporous through-hole membranes (no heat treatment and no ZnO NR growth) are included in Figure 2b,c for comparison purposes. Table 1 details the filtration performance based on the numbers shown in Figure 2.

### 3.3. Filtration Performance of Double-Membrane Filter System 

To further increase the filtration efficiencies of those isoporous through-hole membranes with ZnO NRs, we have measured filtration performances in a double-membrane configuration [21,36,41,42,43]. For this, two separate membranes that grew ZnO NRs were assembled together with a gap of 15 mm by inserting an elastomeric spacer between them. For the double-membrane configuration, we have chosen membranes having ZnO NRs grown from the seed layer annealed at 300 °C. This membrane has shown a rather low-pressure drop (43 Pa), while showing a pretty decent filtration efficiency of 91.5% (Figure 2 and Table 1). The double-membrane filter performances are plotted in Figure 3, and performance values are presented in Table 2. 

The filtration efficiency of the double-membrane system increased up to >97%, while the pressure drop increased slightly to ~80 Pa. Included also in Figure 3 and Table 2 are the filter performances for the single-membrane system (bare and 300 °C annealed ones) and some commercial filters sold in Korea for comparison purposes. Those commercial filters are rated as KF-94 (com #1), KF-80 (com #2), and KF-AD (com #3), respectively. The numbers here mean the filtration efficiency for PM_2.5_, such as >94% for KF-94 and >80% for KF-80. The ‘AD’ denotes ‘anti-droplet’, which is designed for use in the hot summer season and has the capability to prevent droplet/aerosol penetration, which is known as the main route for the infection of SARS-CoV-2. As can be seen in Figure 3 and Table 2, the double-membrane filter system shows much better performance, higher filtration efficiencies (>97%), but a lower pressure drop (~80 Pa), than commercial ones (except the special case of KF-AD, which has a very low filtration efficiency of ~70% with a small pressure drop of ~40 Pa. In fact, this filter performance is comparable to our bare membrane filters shown in Figure 1). It should be noted that there is big difference in the thickness of filters: our membrane filter has a thickness of only ~3 μm, while the commercial ones are very thick, ranging from ~140 μm for KF-AD to ~400 μm for KF-94. Top and cross-sectional SEM images of the commercial filters can be found in Supplementary Figure S5.

### 3.4. Filtration Mechanism of ZnO NR-Grown Membranes

**Figure 4:**
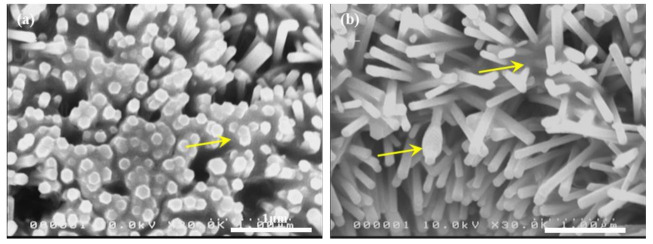
SEM images of membrane surface after filtration, (**a**) top surface and (**b**) sidewall of pore, respectively. Yellow arrows indicate PM deposits on a nanorod in a bead-on-string fashion or in-between multiple nanorods.

Figure 4 shows the SEM images of the membrane surface after PM filtration. Figure 4a shows the top surface of the membrane after the filtration test. The top surface consists of ZnO NRs grown on the isoporous through-hole membrane. As shown, PMs were captured in the space between the NRs. On the surface, the main mechanism for the PM capture is the inertial impaction [36]. The presence of the ZnO NRs increases the available surface area for inertial impaction, and may reduce the escape of recoiled PMs off the membrane surface. Additionally, the fibrous nature of ZnO NRs on the pore sidewall contributes to the capture of PMs by physical interception, as in other fibrous filters.

Figure 4b shows the sidewall of the pore after filtration. PMs are captured on individual nanorods, forming a bead-on-string-like deposit, as indicated by a yellow arrow in Figure 4b. Additionally, there are regions that have PMs captured deep inside the NRs array, also indicated by a yellow arrow. These observations clearly show that the presence of ZnO NRs on the pore sidewall has a beneficial effect on the PM capture. When PM-laden air flows through the pores with ZnO NRs, those NRs play quite a similar role to nanofibers in fibrous filters. In addition to the simple inertial impact, physical interception, Brownian diffusion, and electrostatic adsorption near/at NRs can contribute to the PM capture. Overall, the presence of ZnO NRs on the pore sidewall has enhanced the filtration efficiency without much increase in the pressure drop, when compared to bare membrane filters without ZnO NRs.

### 3.5. Reusability of ZnO NR-Grown Membrane Filters by Cleaning

**Figure 5:**
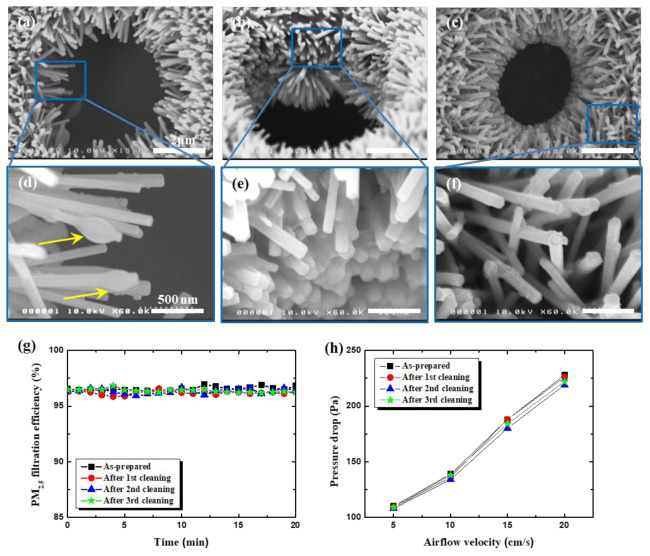
Reusability of ZnO NRs/membrane filters by cleaning with water/ethanol (9:1) spray. SEM images of membrane (single-layer one annealed at 325 °C) surfaces after (**a**) short (5 min) and (**b**) long (20 min) duration of filtration for PM-laden air flow. (**c**) SEM images of membrane surface after the cleaning. (**d**–**f**) Magnified SEM images of membrane surface for (**a**–**c**), respectively. (**g**) Filtration efficiency and (**h**) pressure drop after cycles of filtration (20 min)/cleaning, respectively.

Even with ZnO NRs grown on its surface and pore sidewall, the isoporous through-hole membrane can be reused many times after cleaning, as shown in Figure 5. Initially, as PM-laden air flows through the membranes, the PMs are captured onto a single NR, forming a bead-on-string-like deposit, as shown in Figure 5a (Figure 5d for magnified view). With the filtration time, now the PMs start to fill up the space among NRs, forming a continuous chunk, as shown in Figure 5b and its magnified image in Figure 5e. For the cleaning of these PM-contaminated membrane filters, a water/ethanol (9:1 by volume) mixture was sprayed onto the membrane, rinsed with DI water, and then blow-dried. The sprayed droplets form a bigger drop, and dissolve the PM deposit beneath it. The successful cleaning with the simple spraying lies in the lower surface tension of the solution by adding low surface tension ethanol, which facilitates good wetting of solution drops into deep inside the ZnO NRs array. Figure 5c shows the cleaned ZnO NRs/membrane surface, with a magnified view in Figure 5f, whereby the thick PM deposits were almost completely removed after the cleaning. The reusable filters are very beneficial for the environment and saving resources. The membrane filters were tested for cyclic filtration/cleaning, and the results are shown in Figure 5g,h. As shown, the filter performances, filtration efficiency, and pressure drop do not show any noticeable deterioration upon repeated cycles.

### 3.6. Antibacterial Activity of ZnO NR-Grown Membrane Filters

**Figure 6:**
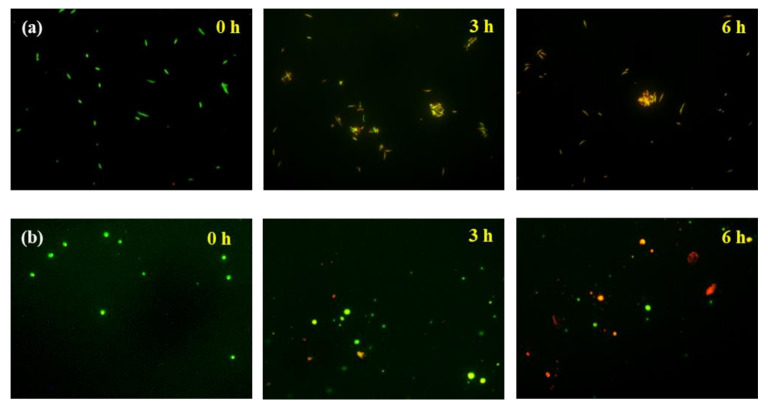
Fluorescence microscopy images of bacterial cells on ZnO NR-grown isoporous through-hole membranes as a function of time. (**a**) Gram-negative *Salmonella enteritidis* (*S. enteritidis*) and (**b**) Gram-positive *Staphylococcus aureus* (*S. aureus*), respectively. The bacterial cells were stained with acridine orange, and green denotes live cells while red denotes dead cells, respectively.

The excellent antibacterial properties of ZnO NRs are visually shown in the fluorescent images of Figure 6. Bacterial colony samples, Gram-negative *S. enteritidis* and Gram-positive *S. aureus*, were grown on agar plates to compare the antibacterial performance of membranes with or without ZnO NRs on membranes. Note here that all the antibacterial tests were performed on membrane seed layers annealed at 325 °C. The bacterial cells were stained with acridine orange, which is a nucleic acid-selective fluorescent dye with cationic properties [44]. Specifically, the dye can permeate the cell membrane and interact with dsDNA and RNA or ssDNA by intercalation and electrostatic attraction, respectively. The live cells emit green fluorescence, while the dead ones emit red fluorescence. As can be seen in Figure 6, the ZnO NR-grown isoporous through-hole membrane exhibits a strong ability of killing bacteria, as manifested in the high portion of dead cells (red color) in 6 h for both Gram-negative and Gram-positive bacteria.

**Figure 7:**
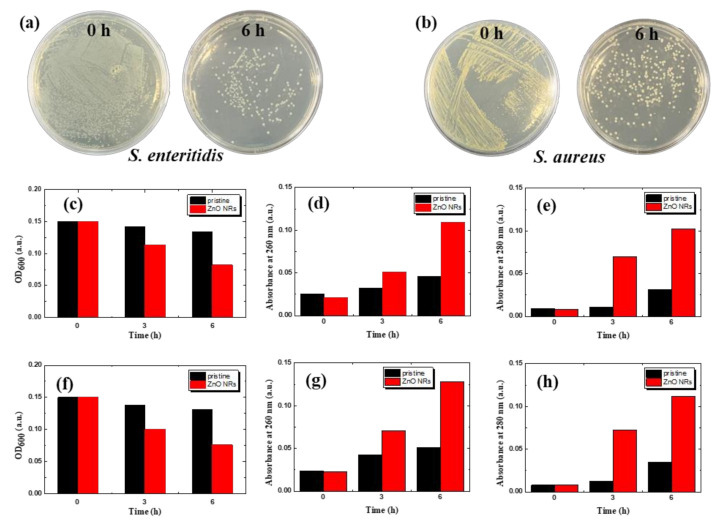
Antibacterial performance of ZnO NR-grown membranes. (**a**,**b**) Photographs of cell culture plates on ZnO NR membranes, for (**a**) Gram-negative *S. enteritidis* and (**b**) Gram-positive *S. aureus*, respectively. (**c**,**f**) Optical density (at 600 nm), (**d**,**g**) absorbance at 260 nm for nucleic acid, and (**e**,**h**) absorbance at 280 nm for proteins, respectively, for each bacterium.

The membrane antibacterial effect was also assessed by counting the number of bacterial colonies, as shown in Figure 7a,b. For both bacteria, the formation of colonies was remarkably suppressed and only a few colonies were observed after 6 h of exposure to ZnO NR-grown isoporous membranes, which indicates a strong antibacterial effect. To monitor the change in live cell concentration, optical densities (OD) (or, absorbance) at various wavelengths were measured. The OD is a very common practice to assess microbial growth in laboratories, and correlates directly with biomass or cell concentration. When the cell membranes are damaged or ruptured by antibacterial activity, nucleic acids and proteins are released, which can be measured by OD at specific wavelengths, 260 nm for nucleic acids and 280 nm for proteins, respectively. Therefore, the antibacterial activity of ZnO NR-grown membranes was monitored by measuring OD at 600, 280, and 260 nm, simultaneously.

Figure 7c,f shows the changes in OD_600_ of *S. enteritidis* and *S. aureus*, respectively, as a function of time. The OD for both bacteria decreased down to ~50% in 6 h of exposure to ZnO NR-grown isoporous membranes, which means almost all the bacteria can be killed in 12 h. At the same time, OD_260_ (Figure 7d,g) for released nucleic acids and OD_280_ (Figure 7e,h) for proteins have shown a concurrent increase with time, which again confirms the antibacterial effect of ZnO NR-grown isoporous membranes. According to the results of previous studies [25], the main antibacterial activity mechanisms of ZnO NRs are classified into three types. First, ZnO, which is in an unstable state in solution, is partially dissolved, releasing Zn^2+^ ions. The released Zn^2+^ ions pass through the bacterial cell wall and accumulate inside the bacterial cell, destroying intracellular components. Second is the mechanical damage to the cell wall. Mechanical damage destroys the cell wall by sharp edges or rough surfaces of the material, resulting in leakage of cell contents and death of bacteria. Lastly, ROS generated by the photocatalytic effect of ZnO NRs under UV-illumination penetrates the bacterial cell wall and destroys components such as intracellular lipids, enzymes, and DNA.

### 3.7. Photocatalytic Activity of ZnO NR-Grown Membrane Filters

**Figure 8:**
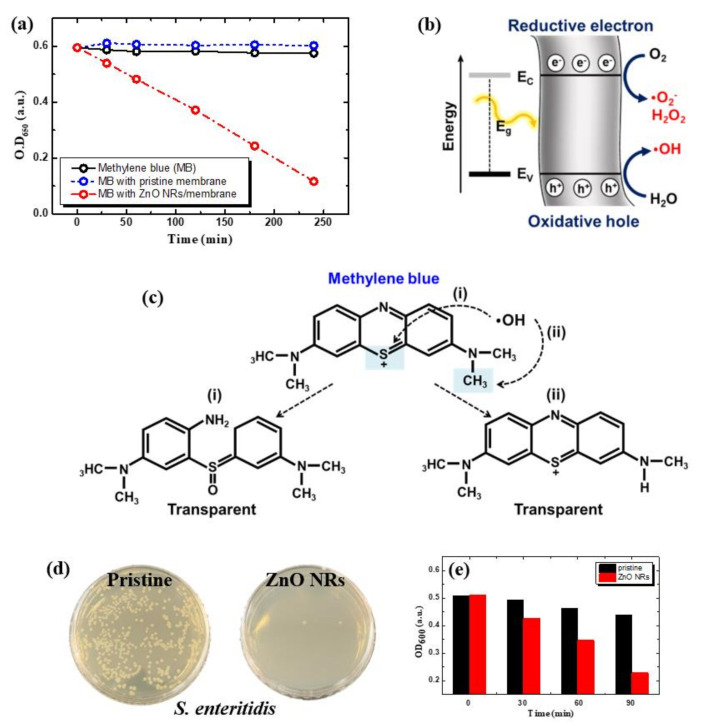
(**a**–**c**) Photocatalytic degradation of methylene blue (MB) by ZnO NRs grown on isoporous membranes under UV-illumination. (**a**) Optical density at 650 nm of MB solution with/without ZnO NRs. (**b**) Energy band diagram of ZnO with oxidative *h*^+^ and reductive *e^−^* to produce hydroxyl radicals, reactive oxygen, and hydrogen peroxide. (**c**) Degradation mechanism of MB by hydroxyl ions. (**d**,**e**) Antibacterial performance of ZnO NR-grown membranes under UV-illumination. (**d**) Photographs of cell culture plates on membranes with and without *S. enteritidis,* and (**e**) optical density at 600 nm.

The photocatalytic activities of the ZnO NR-grown membranes were evaluated for the decomposition of methylene blue (MB). Figure 8a shows the change in OD_650_ for MB solution in pure solution, the bare membrane, and the ZnO NR-grown membrane. The pure MB solution and the solution with the pristine isoporous membrane show constant absorbance as a function of time, which means the concentration of MB remains constant. On the other hand, MB solution with the ZnO NR-grown membrane shows a rapid decrease in OD_650_, denoting photocatalytic decomposition of MB into transparent forms. This is known to be due to ROS generated by the photocatalytic effect of ZnO NRs, as shown in Figure 8b. ZnO is a wide bandgap (~3.37 eV) semiconductor, which produces *e^−^* and *h*^+^ in the valence band (VB) and the conduction band (CB), respectively, upon UV excitation. Oxidative *h*^+^ at the VB and reductive *e^−^* at the CB can oxidize water to hydroxyl radical (OH·) and reduce oxygen to superoxide radical anions (O_2_^−^) and hydrogen peroxide (H_2_O_2_) or hydroperoxyl radicals (HOO·), which can destroy various organic compounds. The formed hydroxyl radical can react with S or methyl group in MB, leading to the formation of a transparent form of MB, as shown in Figure 8c. To demonstrate the antibacterial effect through the mechanism, the change of *S. enteritidis* with and without ZnO NRs on the membrane under UV-illumination was observed (Figure 8d,e). Figure 8d shows that most of the colonies disappeared in the isoporous membrane on which ZnO NRs were grown under UV-illumination for 90 min. This was also confirmed by the optical density graph at 600 nm in Figure 8e. This result shows a much more dramatic change compared to the room light condition in Figure 7 and proves the antibacterial effect of ROS generated by the photocatalytic effect.

## 4. Conclusions

In this work, we have successfully fabricated isoporous through-hole membranes with hydrothermally grown ZnO NRs. The high-temperature (300~375 °C) annealing for seed formation after precursor solution coating could be concurrently used for porosity control of membranes. Thanks to the nanofibrous nature of the grown ZnO NRs, especially on the sidewall of pores, the ZnO NR-grown membranes showed very good filtration performances, such as PM_2.5_ removal efficiency of >97% with a low-pressure drop of ~80 Pa in double-membrane configuration. The ZnO NR-grown membranes can be reused many times after simple cleaning with spraying of a water/ethanol mixture. Further, the grown ZnO NRs endow the membrane with antibacterial and photocatalytic activities. Both Gram-negative (*S. enteritidis*) and Gram-positive (*S. aureus*) bacteria have been found to be killed on the ZnO NR-grown membranes. Upon UV-illumination, the formation of ROS by excited *e*^-^/*h*^+^ in semiconductor ZnO can rapidly decompose MB, which can be used for photocatalytic decomposition of harmful organic compounds during air filtration. The developed membranes can be used in various applications that need an effective PM filtration with antibacterial and photocatalytic functionality.

## Figures and Tables

**Table 1:** Filtration performance of ZnO NR-grown isoporous through-hole membrane filters at an air velocity of 5 cm/s.

Sample	Bare	ZnO 300 °C	ZnO 325 °C	ZnO 350 °C	ZnO 375 °C
η (%)	71.1	91.5	96.5	94.8	97.0
∆P (Pa)	25	43	108	70	102

**Table 2:** Filtration performance for the double-membrane filter system, including single and commercial filters for comparison purposes.

Sample	Bare	ZnO 300 °C	ZnO 300 °C X2	Com #1 (KF94)	Com #2 (KF80)	Com #3 (KF-AD)
η (%)	71.1	91.5	97.5	96.5	86.1	73.0
∆P (Pa)	25	43	83	138	90	40
QF (Pa^−1^)	0.0497	0.0573	0.0442	0.0242	0.0219	0.0327

## Supplementary Materials

The following are available online at https://www.mdpi.com/article/10.3390/nano11123381/s1, Figure S1: Schematic drawing of the experimental set-up for the PM filtration test, Figure S2: SEM images of PDMS stamp and fabricated membranes, Figure S3: Changes in pore size and porosity of isoporous through-hole membranes by annealing temperature with fixing sample edges, Figure S4: Changes in pore size and porosity of isoporous through-hole membranes by annealing temperature without fixing sample edges, Figure S5: SEM images of the commercial filters tested for comparison, Figure S6: Fluorescence microscopy images of bacterial cells on membrane surface without ZnO NRs.
