# Supplementary material for: Recyclable, Antibacterial, Isoporous Through-Hole Membrane Air Filters with Hydrothermally Grown ZnO Nanorods
Source: Nanomaterials (Basel). 2021 Dec 13;11(12):3381. doi: 10.3390/nano11123381 (PMC8707457; doi:10.3390/nano11123381)
Supplement: Supplementary file 1 [file nanomaterials-11-03381-s001.zip › nanomaterials-1481523 supplementary.pdf]

*nanomaterials*

*Supplementary Material*

# Recyclable, Antibacterial, Isoporous Through-Hole Membrane Air Filters with Hydrothermally Grown ZnO Nanorods

**Yong Ho Choi, Moon-Ju Kim, Jia Lee, Jae-Chul Pyun and Dahl-Young Khang ***

Department of Materials Science and Engineering, Yonsei University, Seoul 03722, Korea; yhyhyh825@naver.com (Y.-H.C.); moonjukim@yonsei.ac.kr (M.-J.K.); jia501@naver.com (J.L.); jcpyun@yonsei.ac.kr (J.-C.P.)
* Correspondence: dykhang@yonsei.ac.kr

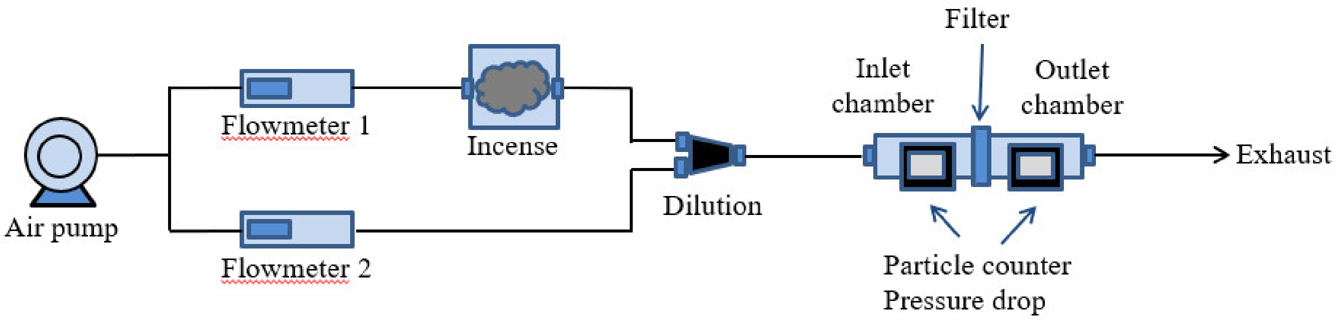


**Figure S1.** Schematic drawing of the experimental set-up for PM filtration test.

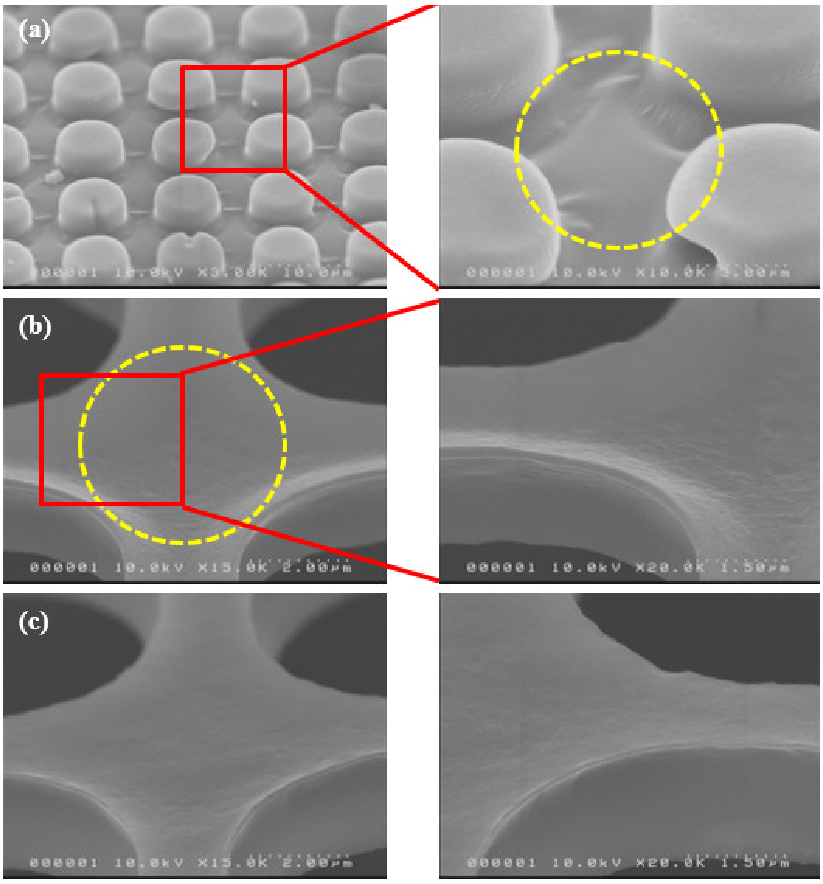


**Figure S2.** SEM images of (**a**) PDMS stamp, (**b**) membrane surface that was in contact with the stamp, and (**c**) the other side of membrane surface, which was not in contact with the stamp during fabrication. As shown, the hump in PDMS

stamp (encircled in yellow in (**a**)) was transferred onto membrane surface. The shallow dip at the diagonal centers between 4 neighboring pores (encircled in yellow in (**b**)) results in thicker seed layer, again which has led to non-uniform growth of ZnO NRs on the membrane surface as shown in Figure 1 of Main Text.

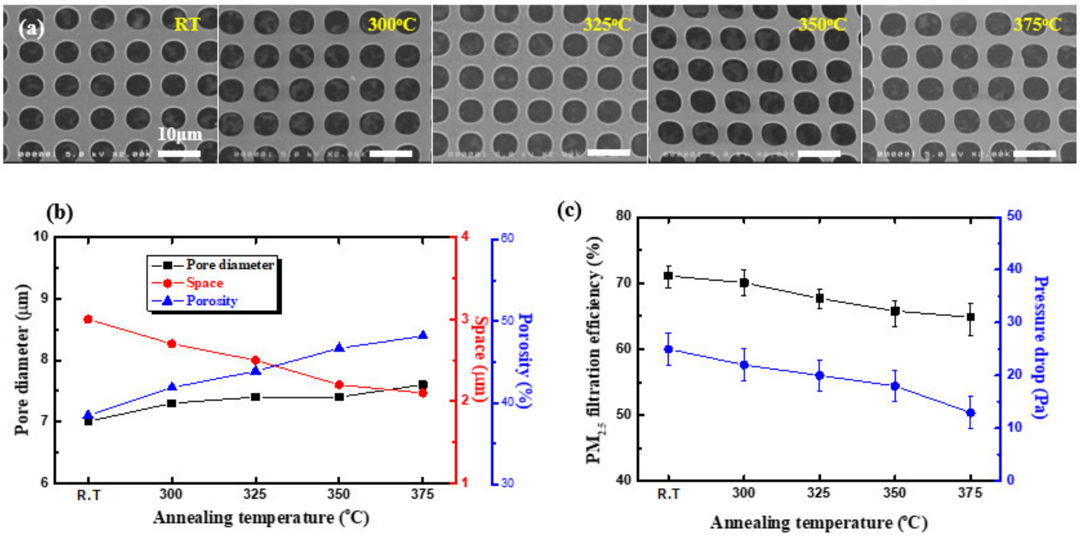


**Figure S3.** Changes in pore size and porosity of isoporous, through-hole membranes by annealing temperature. The membrane samples were annealed with fixing the edges with PI tapes. In this case, the pore diameter increases while the inter-pore spacing decreases due to fixed due to fixed edges of the membrane samples. Thus, the porosity of membranes changes, from ~40% up to ~50%. SEM images of membranes (**a**), and plots for the change in pore size, inter-pore spacing and porosity (**b**,**c**), and PM filtration performance, respectively, of the membranes annealed at different temperatures.

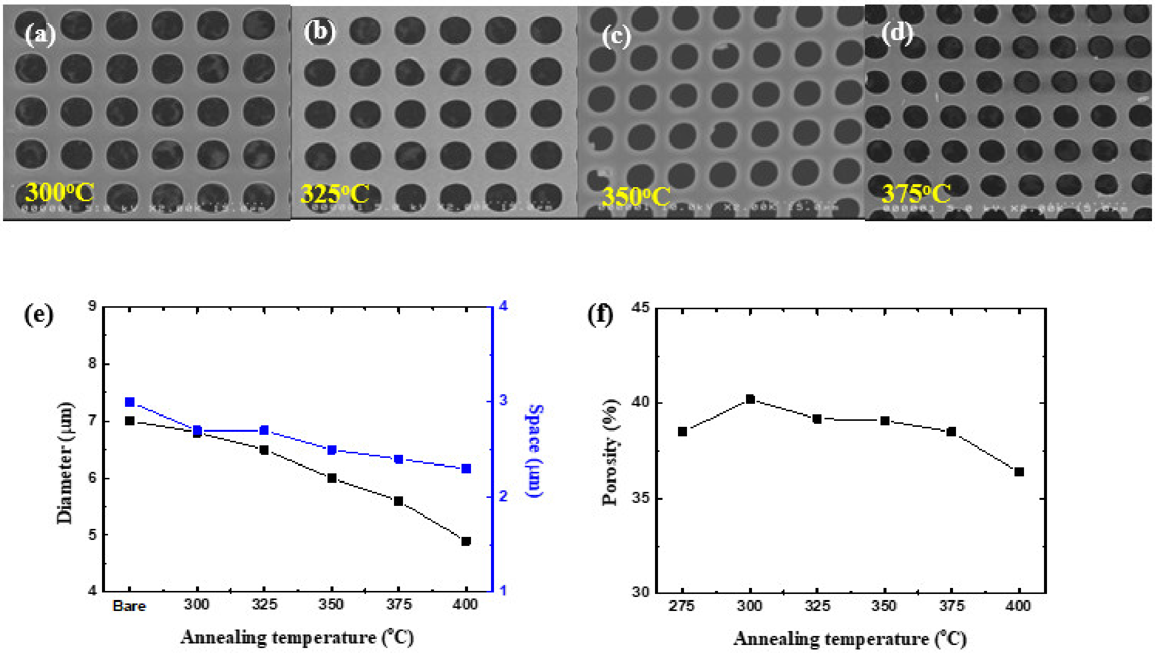


**Figure S4.** Changes in pore size and porosity of isoporous, through-hole membranes by annealing temperature. The membrane samples were annealed without fixing the edges with PI tapes. In this case, the pore diameter and spacing were shrinked simultaneously due to free edges of the membrane samples. Thus, the porosity of membranes d remains almost constant. SEM images (**a**–**d**), pore size and inter-pore spacing (**e**) and porosity (**f**), respectively, of membranes annealed at different temperatures.

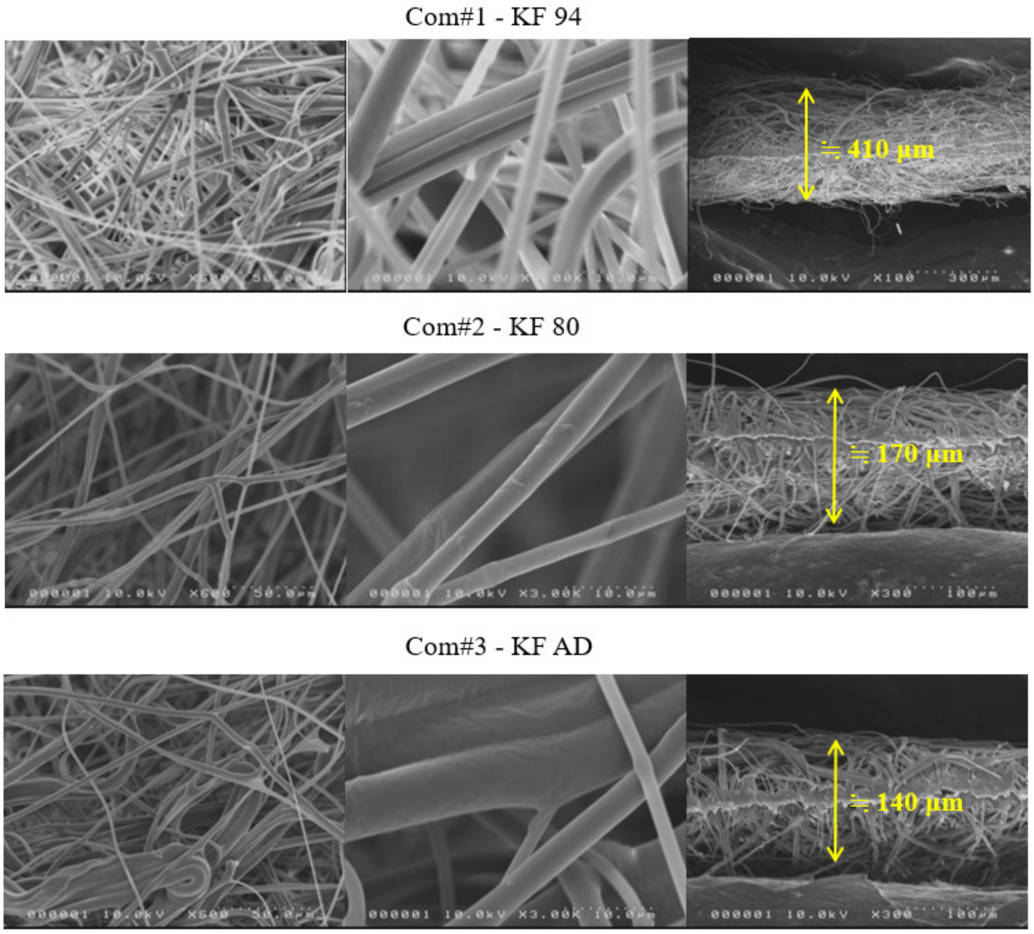


**Figure S5.** SEM images of the commercial filters tested for comparison.

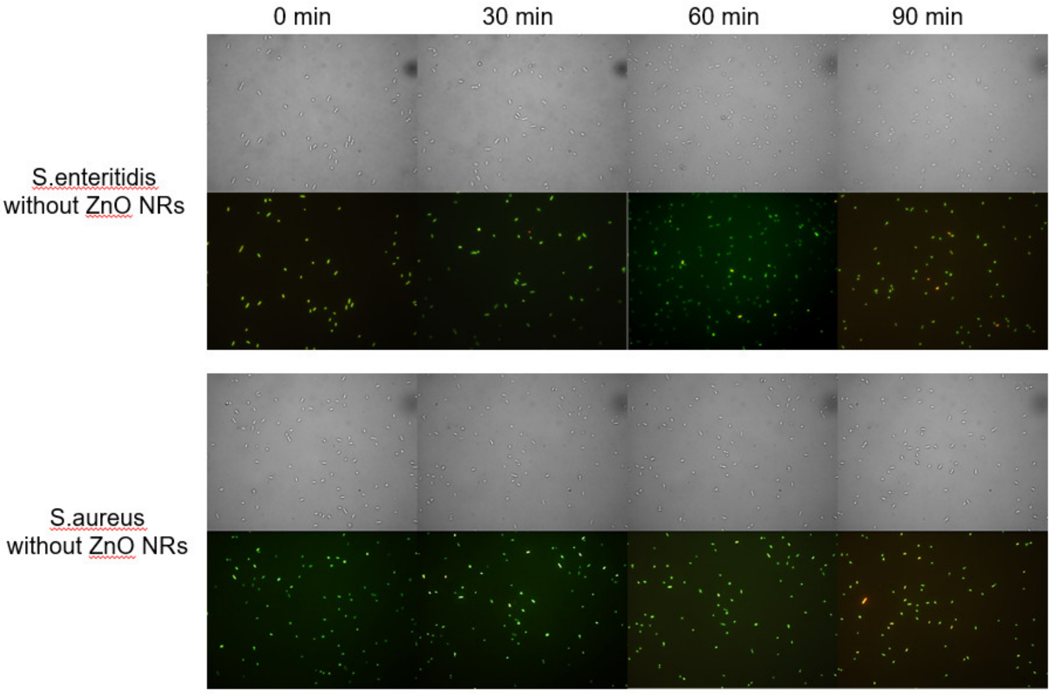


**Figure S6.** Fluorescence microscopy images of bacterial cells on bare isoporous through-hole membranes (without ZnO NRs), as a function of time.
